# Draft Genome Sequence of the Type Strain *Desulfuribacillus alkaliarsenatis* AHT28, an Obligately Anaerobic, Sulfidogenic Bacterium Isolated from Russian Soda Lake Sediments

**DOI:** 10.1128/genomeA.01244-16

**Published:** 2016-11-10

**Authors:** Christopher A. Abin, James T. Hollibaugh

**Affiliations:** a Department of Microbiology, University of Georgia, Athens, Georgia, USA; b Department of Marine Sciences, University of Georgia, Athens, Georgia, USA

## Abstract

*Desulfuribacillus alkaliarsenatis* AHT28^T^ is an obligately anaerobic, sulfur- and arsenate-reducing haloalkaliphile that was isolated from Russian soda lake sediments. Here, we present the 3.1-Mb draft genome sequence for this strain, consisting of 36 contigs with a G+C content of 37.5% and 2,978 protein-coding sequences.

## GENOME ANNOUNCEMENT

Sulfidogenesis is the process by which sulfide is produced through microbial reduction of oxidized sulfur compounds such as sulfate, sulfite, thiosulfate, and elemental sulfur. Elemental sulfur reduction appears to be widespread in alkaline (pH 9 to 11), hypersaline (>3.5% salt) soda lakes, with activities having been observed to surpass that of sulfate reduction under conditions of salt saturation (1). Even though sulfur reduction is a potentially important biogeochemical process in soda lakes, relatively little is known about the phylogeny and physiology of sulfur-respiring haloalkaliphiles. To date, five bacterial and two archaeal strains have been isolated and described (2). Publicly available genome sequences exist for only *Halarsenatibacter silvermanii* SLAS-1^T^ (3, 4) and two strains of *Halanaeroarchaeum sulfurireducens* (5, 6).

*Desulfuribacillus alkaliarsenatis* AHT28^T^ was isolated from a composite of sediment samples collected from six different soda lakes in the Kulunda Steppe, Altai, Russia (7). It is an obligately anaerobic, alkaliphilic, and moderately halotolerant strain with a respiratory metabolism involving the use of elemental sulfur, thiosulfate, or arsenate as electron acceptors. A pure extract of *D. alkaliarsenatis* AHT28^T^ genomic DNA was obtained from the Leibniz Institute German Collection of Microorganisms and Cell Cultures (DSMZ, Braunschweig, Germany) and quantified using a Qubit fluorometer (Thermo-Fisher Scientific/Life Technologies, Waltham, MA, USA). Genome sequencing was performed using the MiSeq platform (Illumina, San Diego, CA, USA) with 250-bp paired-end reads.

A total of 7,186,040 reads were generated, providing about 536-fold median coverage of the genome. The reads were randomly subsampled to an approximate 85-fold coverage with seqtk version 1.0-r63 (https://github.com/lh3/seqtk). Sequence processing and *de novo* assembly was performed using the A5-miseq pipeline (8). The assembly yielded 36 contigs, with maximum and *N*_50_ contig sizes of 689,966 bp and 235,557 bp, respectively. The genome size was 3,106,435 bp with a G+C content of 37.5%, slightly lower than the value of 39.1% previously obtained by the thermal denaturation method (7). The genome was annotated using the RAST server (9), which identified 2,978 protein-coding sequences and 60 tRNA genes. A total of 1,392 coding sequences (47%) were assigned to subsystems. Genome completeness was assessed using AMPHORA2 (10), which confirmed the presence of all 31 phylogenetic marker genes essential in bacteria.

The genome of *D. alkaliarsenatis* AHT28^T^ contained several features associated with an anaerobic respiratory metabolism involving the reduction of sulfur and arsenic compounds. Two operons were found to encode complex iron-sulfur molybdoenzymes related to polysulfide reductase (Psr) and thiosulfate reductase (Phs). Operons encoding a respiratory arsenate reductase (Arr) and a downstream arsenical resistance system (Ars) were also found. Interestingly, the genome encoded a nitrogenase (Nif), suggesting that *D. alkaliarsenatis* AHT28^T^ is capable of converting nitrogen (N_2_) to ammonia (NH_3_). This draft genome will enable further study of bacterial sulfur, arsenic, and nitrogen transformations under haloalkaliphilic conditions.

### Accession number(s).

This whole-genome shotgun project has been deposited at DDBJ/ENA/GenBank under the accession number MIJE00000000. The version described in this paper is the first version, MIJE01000000.
